# The lateral plate mesoderm

**DOI:** 10.1242/dev.175059

**Published:** 2020-06-19

**Authors:** Karin D. Prummel, Susan Nieuwenhuize, Christian Mosimann

**Affiliations:** 1 University of Colorado School of Medicine, Anschutz Medical Campus, Department of Pediatrics, Section of Developmental Biology, 12801 E 17th Avenue, Aurora, CO 80045, USA; 2 Department of Molecular Life Sciences, University of Zürich, Winterthurerstrasse 190, 8057 Zürich, Switzerland

**Keywords:** Lateral plate mesoderm, Cardiovascular system, Cell fate, Development, Evolution, Gene regulation

## Abstract

The lateral plate mesoderm (LPM) forms the progenitor cells that constitute the heart and cardiovascular system, blood, kidneys, smooth muscle lineage and limb skeleton in the developing vertebrate embryo. Despite this central role in development and evolution, the LPM remains challenging to study and to delineate, owing to its lineage complexity and lack of a concise genetic definition. Here, we outline the processes that govern LPM specification, organization, its cell fates and the inferred evolutionary trajectories of LPM-derived tissues. Finally, we discuss the development of seemingly disparate organ systems that share a common LPM origin.

## Abstract (precis)

**Summary:** The lateral plate mesoderm is the origin of several major cell types and organ systems in the vertebrate body plan. How this mesoderm territory emerges and partitions into its downstream fates provides clues about vertebrate development and evolution.

## Introduction

During gastrulation in vertebrates, the mesoderm forms axial, paraxial and lateral domains that harbor precursor cells for distinct organ systems. The lateral plate mesoderm (LPM) condenses into bilateral sheets of cells at the lateral edge of the developing vertebrate embryo, classically referred to as the lateral plate. While clearly discernible after gastrulation, the dynamic nature of the LPM is challenging to visualize and track during earlier development. Moreover, fate maps derived from various model organisms provide seemingly conflicting data, in part due to differences in lineage-tracing techniques and readouts (Lane and Smith, 1999), as well as uneven nomenclature to describe the LPM as ventral mesoderm, leading-edge mesoderm, visceral mesoderm, ventrolateral mesoderm or lateral mesoderm.

**Figure:**
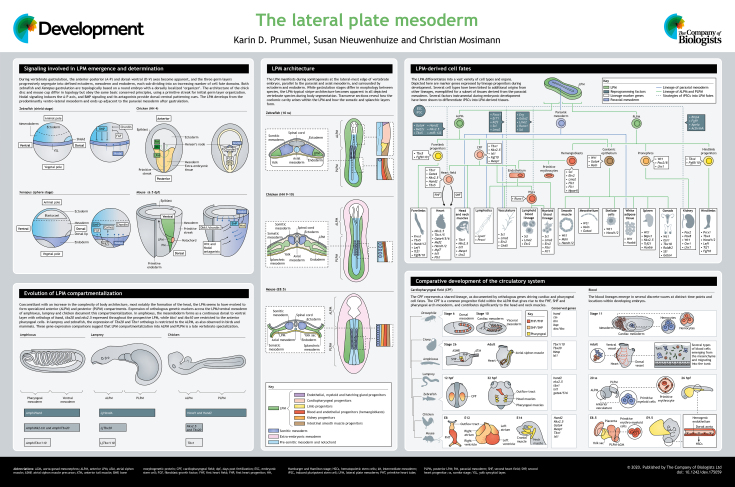

The LPM also develops into a bewildering array of downstream cell fates. Lineage maps derived from transplantation and cell labeling experiments have linked the LPM to the origin of cardiovascular, hematopoietic, kidney, smooth muscle, craniofacial (head/neck) muscle, mesothelial and limb connective tissue progenitors (Lane and Smith, 1999; Selleck and Stern, 1991; Warga and Nüsslein-Volhard, 1999). While these cell fates contribute essential structures to the adult vertebrate body, their earlier developmental connection is not immediately apparent from the final functional organs.

Recent breakthroughs in genetic lineage tracing, reporter assays, live imaging and single-cell RNA-sequencing continue to uncover new details of the LPM and its derivatives, detailing its substantial contribution to the evolution of the vertebrate body plan. Here, we outline how the LPM emerges within the embryo and summarize the latest insights into how the LPM generates its diverse interconnected cell fates.

## LPM specification and organization

Although the characteristic stripe architecture of the LPM becomes apparent during the segmentation stages, the LPM originates during gastrulation together with the axial and paraxial mesoderm between ectoderm and endoderm (Davidson and Zon, 2004; Lawson et al., 1991; Rosenquist, 1970; Tam and Beddington, 1987). Prominent signaling cascades influencing early LPM formation include the bone morphogenetic protein (BMP) and Nodal pathways that coordinate the patterning of anterior-posterior (A-P) and dorso-ventral axes (Arnold and Robertson, 2009; Hill, 2018; Martinez-Arias and Steventon, 2018). High levels of BMP signaling in the ventral domain of the embryo chiefly specifies the mesoderm territory that forms the LPM in all vertebrates (Ferretti and Hadjantonakis, 2019; Nishimatsu and Thomsen, 1998). Nonetheless, coinciding with Nodal activity, LPM-assigned cell fates also emerge along the marginal zone of *Xenopus* and zebrafish embryos that contribute the cells at the circumference of the forming embryo (Lane and Smith, 1999; Prummel et al., 2019; Schier and Talbot, 2005; Warga and Nüsslein-Volhard, 1999). Prominently shown in zebrafish embryos, an increasing range or activity of BMP signaling in ventralized mutants results in larger domains of LPM-expressed genes for erythrocyte, pronephros and vascular lineages (Hammerschmidt et al., 1996; Mullins et al., 1996; Sidi et al., 2003). Conversely, dorsalizing mutations that affect BMP ligands, BMP regulators or the loss of ventro-posterior transcription factors, such as Cdx4, cause the loss of posterior LPM structures (Davidson et al., 2003; Hild et al., 1999; Kishimoto et al., 1997; Mullins et al., 1996; Nguyen et al., 1998; Ro and Dawid, 2009; Schier and Talbot, 2001). Nonetheless, BMP and Nodal are not instructive to trigger LPM formation per se (Xu et al., 2014), hinting at a more-complex signaling interplay leading to LPM induction. Fibroblast growth factor (FGF), canonical Wnt and retinoic acid (RA) signaling also influence the emerging LPM domains (Furthauer et al., 2004; Holley and Ferguson, 1997; Rossant and Tam, 2009; Schier and Talbot, 2005), including the specification of the heart field (Deimling and Drysdale, 2011; Gessert et al., 2010; Itoh et al., 2016; Lengerke et al., 2011; Wang et al., 2019a) or of kidney and blood progenitors (Duester, 2008; Lasagni et al., 2015; Naylor et al., 2016; Niederreither and Dollé, 2008).

Following gastrulation, the LPM takes on its characteristic architecture: bilateral stripes (‘plates’) of LPM progenitors form laterally in the embryo and subsequently partition along the A-P and medial-to-lateral axes into dedicated cell fate domains (Gurdon, 1995; Kessler and Melton, 1994; McDole et al., 2018; Prummel et al., 2019). Convergent extension of the embryo axis involving planar cell polarity (PCP) signaling also affects the final position of the LPM adjacent to the forming somites (paraxial mesoderm) and in relation to the ectoderm and the endoderm (Erter et al., 2001; Heisenberg and Solnica-Krezel, 2008; Saykali et al., 2019); however, LPM-specific mechanisms of cell arrangement have yet to be described in detail. In amniotes, the post-gastrulation LPM splits into a dorsal somatic layer and a ventral splanchnopleuric layer. During body segmentation, the LPM further separates into distinct anterior (ALPM) and posterior (PLPM) domains, while the bilateral cell fields gradually differentiate into descendant cell fates with distinct gene expression patterns.

### Lineage markers in the LPM

The diverse temporal, spatial and combinatorial activities of the signaling pathways involved in LPM specification make their exact influence challenging to dissect. Furthermore, the lack of a concise genetic or molecular definition of the LPM has limited its description. The bulk of our understanding of LPM fates derives from the regionalized, post-gastrulation expression patterns of individual transcription factor genes. Most prominently, the expression of (and transgenes based on) *Foxf1*, *Bmp4*, *Hoxb6*, *Hand1*, *Hand2*, *Gata4* and *Prrx1* have been harnessed in mouse and chick embryos to track various aspects of LPM patterning (Becker et al., 1996; Firulli et al., 1998; Martin and Olson, 2000; Ormestad et al., 2004; Rojas et al., 2005). However, these gene expression domains are either broader than, or delineate only parts of, the entire LPM. While expression of the T-box factor brachyury is commonly used as reference for mesodermal lineages in mammals (Huber et al., 2004; Loh et al., 2016; Technau and Scholz, 2003), expression and activity of brachyury is dispensable for LPM formation in a variety of animal models, mainly contributing to tail formation and its ancestral role in notochord development (Clements et al., 1996; Gurdon, 1995; Halpern et al., 1993; José-Edwards et al., 2015; Schulte-Merker et al., 1994; Wilkinson et al., 1990).

Conversely, the +2.0 kb enhancer in the zebrafish *draculin* (*drl*) locus (*+2.0drl*) is specifically active in LPM-primed mesendoderm during zebrafish development by responding to the mesendoderm regulators eomesodermin A, FoxH1 and Mixl1, together with BMP- and Nodal-controlled Smads (Prummel et al., 2019). Although *drl* seems to be a zebrafish-specific zinc-finger gene, *+2.0drl* enhancer-based reporter transgenes also label the emerging LPM in chick, axolotl and lamprey, and in the nonvertebrate chordates *Ciona* and amphioxus, suggesting that LPM emergence is instructed by a conserved molecular program (Prummel et al., 2019). It remains to be determined whether LPM formation is universally controlled by eomesodermin A, FoxH1 and Mixl1 orthologs across chordates, which mechanisms induce expression of the genes that pattern the LPM post-gastrulation, and whether any of these conserved genes are LPM specific.

### Evolution of the LPM

The seemingly complex developmental relationship of the final organs of the LPM becomes more accessible in the light of their evolutionary connections. In amphioxus, the mesendoderm forms as a continuous dorsal to ventral layer, and a LPM-resembling domain can be recognized in between the dorsal somites and the ventral endoderm (Bailey and Miller, 1921; Bertrand et al., 2011b; Holland, 2018; Onimaru et al., 2011). This seemingly simple setup could hint at the original mesendoderm architecture in the last common chordate ancestor (Kozmik et al., 2001), which is set up by the conserved LPM-instructing program (Prummel et al., 2019). Amphioxus orthologs of genes expressed in the LPM in vertebrates, such as *Foxf1*, Hand genes, *Tbx20* and *Nkx2.5*, are active throughout the whole ventrolaterally located mesoderm, indicating that the LPM in amphioxus does not segregate into ALPM and PLPM, whereas ALPM and PLPM are clear features in lampreys (Onimaru et al., 2011; Tanaka, 2016a). These observations suggest that LPM compartmentalization along the embryonic axes is a vertebrate adaptation. Curiously, in *Drosophila*, visceral mesoderm formation depends on Bap and the Foxf1 ortholog Bin, whereas heart formation relies on GATA factors together with Hand and Tin, the orthologs of the vertebrate heart regulators Hand1, Hand2 and Nkx2.5 (Azpiazu and Frasch, 1993; Bodmer, 1993; Zaffran et al., 2001). This conservation of LPM-associated gene expression hints at a deeply rooted molecular LPM program dating back to early bilaterians.

## From stripes to organs: development of LPM-derived structures

### Cardiovascular system

The formation of the circulatory system provides an illustrative example for LPM-derived organ development. During early somitogenesis, the heart forming in the ALPM and the endothelial and hematopoietic lineages forming in both ALPM and PLPM become detectable by both shared and specialized gene expression patterns. In the ALPM, endocardial and myocardial progenitor populations become detectable adjacent to cranial endothelial progenitors by expression of *Nkx2.5*, *Etv2*, *Lmo2* and *Scl*/*Tal1*, whereas the PLPM harbors the trunk endothelial and primitive erythrocyte progenitors also expressing *Etv2*, *Lmo2*, *Scl*/*Tal1* and *Gata1*, but not *Nkx2.5* (Bussmann et al., 2007; Davidson and Zon, 2004; Loh et al., 2016; Scialdone et al., 2016; Tremblay et al., 2018; Vincent and Buckingham, 2010).

### Heart

In the emerging mouse mesendoderm, *Mesp1* expression downstream of *Eomes* demarcates the earliest cardiac progenitors (Costello et al., 2011; Kitajima et al., 2000; Saga et al., 2000) that upregulate *Gata4*, *Nkx2.5* and *Hand2* (Bondue et al., 2008; Kelly et al., 2014). This initial requirement for Mesp factors holds true for cardiac progenitor formation in *Ciona* (Satou et al., 2004). In contrast, Mesp1 orthologs in *Drosophila* and in zebrafish seem dispensable for cardiogenesis (Deshwar et al., 2016; Moore et al., 2000; Yabe et al., 2016). These peculiar findings suggest a degree of flexibility in cardiac progenitor initiation, which has yet to be further characterized. Curiously, akin to *Eomes*, *Smarcd3* (*BAF60c*) expression in the mouse precedes *Mesp1* upregulation and is essential for heart formation (Lickert et al., 2004). Regulatory elements from the mouse *Smarcd3* locus actively drive reporter expression in the zebrafish ALPM (Yuan et al., 2018), while zebrafish Smarcd3 function has been linked to paraxial muscle differentiation through interaction with brachyury/Ntl (Ochi et al., 2008). These data might indicate that Smarcd3 orthologs act as co-factors to T-box factors such as Eomes or other yet-to-be-determined transcription factors. How universal this interplay is for cardiac progenitor formation or within the LPM in general warrants further investigation.

The subsequent migration of cardiac progenitors to the midline and the formation of the linear heart tube depends on several factors, including platelet-derived growth factor (PDGF) and Robo-Slit signaling providing extrinsic and intrinsic migration cues (Bloomekatz et al., 2017; Fish et al., 2011; Qian et al., 2005; Zhao and Mommersteeg, 2018). In zebrafish (*sox32/cas*) and mouse (*Sox17*) endoderm mutants, multiple heart tubes form within the bilateral ALPM, indicating that cardiac progenitors have an intrinsic propensity to form a rudimentary heart (Alexander et al., 1999; Dickmeis et al., 2001; Kanai-Azuma et al., 2002; Kikuchi et al., 2001; Lickert et al., 2002). As a universal trait, the developing heart incorporates cells from two ALPM-associated progenitor fields, the so-called first versus second heart fields (FHF and SHF, respectively) (Abu-Issa and Kirby, 2008; Meilhac et al., 2004; Stolfi et al., 2010; Tirosh-Finkel et al., 2006). While the FHF descendants set up the initial heart tube with atrium and ventricle for systemic circulation, the addition of SHF progenitors extends the heart on both poles (de Pater et al., 2009; Felker et al., 2018; Grimes and Kirby, 2009; Hami et al., 2011; Lazic and Scott, 2011; Zhou et al., 2011). As a fundamental building block of all vertebrate hearts, the interplay of FHF and SHF influences cardiac conductivity and facilitates sequential contraction (Mosimann et al., 2015); however, why two progenitor pools are required for heart formation remains uncertain. SHF descendants contribute to the increasingly complex compartmentalization in the heart of terrestrial vertebrates, culminating in a right ventricle that is dedicated to pulmonary circulation (Kelly, 2012; Koshiba-Takeuchi et al., 2009; Swedlund and Lescroart, 2019; Vincent and Buckingham, 2010).

### Endothelium

In vertebrates, blood and endothelium form concomitantly with the heart. While expressing an overlapping set of genes, endothelial and hematopoietic progenitors in ALPM and PLPM develop seemingly disconnected from each other but temporally in sync. Endothelium and blood arise, at least partially, from bipotent hemangioblasts, as well as from fate-restricted angioblasts and hematopoietic progenitors (Choi et al., 1998; Murray, 1932; Sabin, 1917; Vogeli et al., 2006). The zebrafish *npas4l*/*cloche* mutant is virtually devoid of blood and endothelium (with exception of few surviving angioblasts), as evident by the broad lack of *scl*, *lmo2* and *etv2* expression (Marass et al., 2019; Reischauer et al., 2016; Stainier et al., 1995). While a clear functional Npas4l ortholog is currently unknown outside of fishes, these findings place Npas4l at the top of the developmental hierarchy controlling the formation of endothelial/hematopoietic progenitors. Among the earliest conserved endothelial/hematopoietic transcription factors is the ETS factor Etv2 that, together with Scl/Tal1, governs endothelial/hematopoietic and hemangioblast formation in mouse, chick and zebrafish (Craig and Sumanas, 2016; Oh et al., 2015). With over a dozen family members expressed at different developmental time points downstream of Etv2, ETS factors play a continued role in endothelial differentiation towards a functional vascular network with veins and arteries. For example, the expression of Fli1, Erg and Ets1 provides powerful endothelial markers in various model systems (Craig and Sumanas, 2016). In addition to transcription factors, vascular-endothelial growth factor (VEGF) signaling is guiding endothelial differentiation (Simons et al., 2016). Reflecting this role, VEGF receptor genes such as *Vegfr2* and *Flk1* and their paralogs are among the earliest genes contributing to hemangioblast formation (Chung et al., 2002; Ema et al., 2003; Loh et al., 2016; Thompson et al., 1998). Nonetheless, despite a wealth of insights into the mechanisms of vascular system formation, how Etv2 and its related factors (and the even more upstream-acting Npas4l in zebrafish) are selectively activated within the cardiovascular-primed LPM remains unknown.

### Blood

Blood emerges in several discrete waves of hematopoiesis at distinct time points and locations within the embryo, and its development is closely intertwined with endothelium formation (Davidson and Zon, 2004; Orkin and Zon, 2008). In teleosts and amphibians, a specialized primitive wave of myeloid progenitors emerges in the ALPM that might be an ancestral trait (Davidson and Zon, 2004; Herbomel et al., 1999; Ohinata et al., 1990). The first primitive wave of PLPM-derived blood consists of transient, embryonic erythrocytes that stem from Scl/Tal1-, Lmo2- and Gata1-expressing progenitors (Davidson and Zon, 2004; Mead et al., 2001; Orkin and Zon, 2008). An intermediate wave of erythro-myeloid progenitors form encased within the developing vessels in zebrafish (Bertrand et al., 2007), and several yolk sack and placenta cell populations have been attributed with intermittent hematopoietic potential in mice (Orkin and Zon, 2008; Palis et al., 1999; Zhang et al., 2018). Finally, Runx1-expressing definitive hematopoietic stem cell (HSC) progenitors bud off from the ventral wall of the dorsal aorta (so-called hemogenic endothelium) through an endothelial-to-hematopoietic transition in zebrafish (Bertrand et al., 2010; Kissa and Herbomel, 2010) and in mice (Boisset et al., 2010; Zovein et al., 2008), while a somite-based contribution of aortic wall and HSCs has also been reported in zebrafish (Qiu et al., 2016). In addition to hemangioblasts, the repeated interdependence of hematopoietic waves on endothelial cells possibly hints at a joint evolutionary origin (Pascual-Anaya et al., 2013). Such scenarios have received further support from the ontogeny of macrophage lineages (Sanz-Morejón et al., 2019; Shigeta et al., 2019) and from observations made in a variety of invertebrates (Cloney, 1982; Cloney and Grimm, 1970; Hartenstein and Mandal, 2006; Monahan-Earley et al., 2013; Munoz-Chapuli, 2011; Munoz-Chapuli et al., 2005; Scimone et al., 2018; Shida et al., 2003). Together with the joint expression of key genes in endothelial and hematopoietic progenitors, a common origin of all cardiovascular lineages within the LPM provides the developmental context to tie these interdependent cell fates together.

### Lymphatics

Related to endothelium and blood, the origin of lymphatic vessels seems more complex. While trunk and cardiac lymphatics have been shown to originate from LPM-derived lineages, in particular from veins in mouse and zebrafish (Lioux et al., 2020; Maruyama et al., 2019; Nicenboim et al., 2015; Semo et al., 2016), recent work in the mouse indicates that *Pax3:Cre*-expressing paraxial mesoderm is a major source of trunk and cardiac lymphatic vessels (Stone and Stainier, 2019). Similarly, *Pax3:Cre*-expressing cells as paraxial mesoderm contribute to at least parts of the endothelium in the mouse forelimb (He et al., 2003; Huang et al., 2003; Hutcheson et al., 2009; Mayeuf-Louchart et al., 2014; Pardanaud et al., 1996; Pouget et al., 2006; Yvernogeau et al., 2019). Whether *Pax3*-based lineage tracing in these scenarios is strictly paraxial mesoderm specific (Engleka et al., 2005), and whether a non-LPM origin for lymphatics and individual endothelial lineages is a universal trait across vertebrates, remain to be elucidated.

### Craniofacial muscle lineages

In line with the surprising lineage diversity of the LPM, detailed lineage tracing studies in the mouse have revealed that the ALPM progenitors that form SHF also contribute to neck and craniofacial muscles alongside paraxial mesoderm and neural crest contributions to their connective tissue (Bothe and Dietrich, 2006; Lescroart et al., 2010; Meilhac et al., 2004; Nathan et al., 2008; Tirosh-Finkel et al., 2006). Although the nomenclature of mesodermal domains in the developing head suffers from disparities across the literature, at least part of the cardiopharyngeal field (CPF) designates an ALPM-centered progenitor pool that, in addition to forming the heart, also contributes to craniofacial and neck muscles (Diogo et al., 2015). Comparative anatomical and genetic studies across chordates have demonstrated joint cardiac and branchiomeric muscle formation, together with overlapping expression patterns in the anterior mesoderm of key regulators, including *Nkx2.5*, *Isl1* and *Tbx1* (Diogo et al., 2015; Felker et al., 2018; Gopalakrishnan et al., 2015; Heude et al., 2018; Lescroart et al., 2015; Michailovici et al., 2015; Paffett-Lugassy et al., 2017; Stolfi et al., 2010; Wang et al., 2019b). The evolutionary timeline of additional cranial muscle groups that are distinct from trunk muscle trajectories coincides with the adaption of multi-chambered hearts (Comai et al., 2019; Diogo et al., 2015; Heude et al., 2018; Theis et al., 2010). The first traces of a CPF are even detectable in cephalochordates, by the expression of several T-box transcription factors, including *Tbx1*/*10* and *Tbx20* in the ventrolaterally located mesoderm of amphioxus (Holland et al., 2008; Onimaru et al., 2011). More insights into the CPF promises to reveal new insights into vertebrate head and neck evolution, and how seemingly disparate mesodermal populations in the head, such as the cephalic or cranial paraxial mesoderm, are interconnected (Bertrand et al., 2011a).

### Kidney

In amniotes, the kidney develops in a distinct temporal sequence via three different stages: pronephros, mesonephros and metanephros (adult kidney). In teleosts and amphibians, the mesonephros functions as the adult kidney. The kidney primordia emerge as bilateral fields expressing *Wt1*, *Lhx1*, *Pax2* and *Pax8* in the PLPM during early somitogenesis (Heller and Brändli, 1999; Mudumana et al., 2008; Nelson et al., 2014; Serluca and Fishman, 2001; Tena et al., 2007; Terashima et al., 2014). During differentiation, besides the rostrally positioned glomerulus, the nephric tubule epithelium is specified along the A-P axis in several segments (McMahon, 2016; Naylor et al., 2016; Serluca and Fishman, 2001). Curiously, the mediolateral position of kidney progenitors relative to the paraxial mesoderm is distinct between fish and amniotes: in amniotes, kidney progenitors form directly adjacent to the paraxial somites as the most medially located LPM stripe (also referred to as intermediate mesoderm). Conversely, in fish, kidney progenitors form lateral to the endothelium-hematopoietic progenitors in the LPM, clearly embedding kidney origins in the context of LPM formation.

### Limb skeleton and connective tissue

A powerful example of the evolutionary adaptability of the LPM is the connective tissue and skeleton of the paired appendages, which has been repeatedly reviewed (Hiscock et al., 2017; Petit et al., 2017; Zeller et al., 2009). Fore- and hindlimb buds emerge from the somatic LPM at specific positions along the A-P axis. RA signaling and Hox genes are involved in properly positioning the progenitor fields (Moreau et al., 2019). In close interplay with the ectoderm secreting FGF ligands (i.e. Fgf8), LPM-expressed *Tbx5* and *Tbx4*, among other factors, contribute to initiating limb formation in the ALPM and PLPM, respectively (Bruneau et al., 2001; Koshiba-Takeuchi et al., 2009; Minguillon et al., 2012; Nishimoto and Logan, 2016; Rallis et al., 2003). The limb skeleton and connective tissue have been predominantly fate-mapped to an LPM origin, while limb musculature is contributed by paraxial/somitic mesoderm (Nishimoto and Logan, 2016; Tanaka, 2016b). Curiously, *Tbx5* is required for both forelimb and heart development, and its expression encompasses the forelimb and heart field progenitors that emerge adjacently within the LPM (Bickley and Logan, 2014; Rallis et al., 2003). Whether *Tbx5* expression is driven by a joint program in both progenitors or if each progenitor field responds to separate inputs remains uncertain (Minguillon et al., 2012).

### Coelomic epithelium and associated structures

The epithelial lining of the coelomic cavities, the so-called mesothelium, is also LPM derived. The mesothelium covers the body cavities (parietal layers) and the organs within (visceral layers), which have been linked to somatic and splanchnic LPM origins, respectively (Mutsaers and Wilkosz, 2007). In mice, *Wt1* and *Gata4*, as well as *Msln* expression, provide the earliest (E9.0) genetic and lineage markers for the visceral coelomic epithelium, which is detectable lateral to the urogenital progenitors (Ariza et al., 2016; Armstrong et al., 1993; Cano et al., 2013; Chau et al., 2014; Delgado et al., 2014; Que et al., 2008; Rinkevich et al., 2012). The coelomic epithelium is increasingly recognized as the source of a wide range of cell types (Ariza et al., 2016). In particular, smooth muscles surrounding the gastrointestinal and respiratory tract, and the vascular system, as well as additional fibroblast-like lineages, have been tracked in mice (Ariza et al., 2018; Asahina et al., 2011; Cano et al., 2013; Carmona et al., 2013; Chau et al., 2011; Ijpenberg et al., 2007; Pérez-Pomares et al., 2004; Que et al., 2008; Rinkevich et al., 2012; Sinha and Santoro, 2018; Wilm et al., 2005) and chick (Winters et al., 2012). Genetic lineage tracing in zebrafish further supports a conserved LPM origin for the visceral intestinal smooth muscles (Gays et al., 2017). Nonetheless, which smooth muscle lineages (or if all) feature a LPM origin remains unresolved.

The spleen develops from condensations of the Wt1-positive coelomic epithelium in mice and humans (Burn et al., 2008; Endo et al., 2015; Hecksher-Sorensen et al., 2004), and visceral white adipose tissue (WAT) depots in mice have been suggested to derive from LPM, as indicated by genetic lineage tracing with *Wt1:creERT2* and *HoxB6:creERT2* (Chau et al., 2014; Krueger et al., 2014; Sanchez-Gurmaches et al., 2015; Sebo et al., 2018; Zhou et al., 2008). Finally, gonadal structures have been assigned to have a common origin in the adrenogonadal primordium, which in mouse and rat arises from a thickening of the coelomic epithelium (Hatano et al., 1996; Ikeda et al., 1994). In these models, *Gata4*, *Tbx18*, *Tcf21* and *Wt1* are among the earliest activated genes expressed throughout the genital ridge, recapitulating a gene expression signature observed in other regions of the coelomic epithelium (Airik et al., 2006; Bohnenpoll et al., 2013; Cui et al., 2004; Häfner et al., 2015; Hammes et al., 2001; Hu et al., 2013; Karl and Capel, 1998; Liu et al., 2015). Lineage tracing in mouse with *Cre* driver lines including *Wt1:creERT2* (Liu et al., 2015) and *Tbx18:creERT2* (Bohnenpoll et al., 2013) have shown that coelomic epithelium-derived precursor cells give rise to several cell types within the primitive gonads, such as the Sertoli cells in the testis and the follicular cells in the ovary. During gonadal differentiation, the primordial germ cells (PGCs) arrive at the genital ridge and are retained within the forming gonads (Barton et al., 2016). Despite these fascinating links to LPM origins, how the initial mesothelium-associated lineages arise within the LPM remains to be charted.

## Concluding remarks

The LPM connects diverse organ systems in the vertebrate body plan. How individual LPM-derived lineages emerge during development is of major interest for controlled stem cell reprogramming. Two principal approaches promise the generation of therapeutically relevant, human LPM derivatives. First, direct reprogramming into specified LPM fates has been achieved with defined combinations of developmental transcription factors in iPSCs, ESCs and somatic cells, as exemplified with the programming of fibroblasts into beating cardiomyocytes using *GATA4*, *HAND2*, *MEF2C* and *TBX5* (Sadahiro et al., 2015; Song et al., 2012; Takasato et al., 2014). Second, the stepwise recapitulation of developmental signaling towards a LPM gene expression signature can be achieved in ES cells, as demonstrated with timed exposure to TGFβ and BMP (Loh et al., 2016; Mendjan et al., 2014). Nonetheless, characterizing and categorizing LPM-derived cells through reprogramming still relies on limited marker signatures (Loh et al., 2016; Mendjan et al., 2014; Orkin and Zon, 2008; Takasato and Little, 2015). Nonetheless, LPM-based organoid models promise to provide a potent source of clinically relevant cell types and new platforms to probe the basic mechanisms of LPM patterning (Holloway et al., 2019). Together, these new and exciting models enable elucidation of the mechanisms driving LPM emergence from embryonic mesendoderm. Easily rivaling the cell fate potential of the neural crest (Mayor and Theveneau, 2013), we are slowly unraveling the cellular properties that render the LPM capable of forming its diverse descendant cell fates and organ systems.
